# Emergency Department Presentation of a Patient with a Severe Upper Gastrointestinal Bleed: A Simulation Case for Training Emergency Medicine Residents

**DOI:** 10.7759/cureus.3908

**Published:** 2019-01-17

**Authors:** Kerry-Lynn Williams, Cody Dunne, Michael H Parsons

**Affiliations:** 1 Medical Education and Simulation, Memorial University of Newfoundland, St. John's, CAN; 2 Emergency Medicine, Memorial University of Newfoundland, St. John's, CAN

**Keywords:** simulation, emergency medicine, medical education, simulation based medical education, hemmorhage, shock, upper gi bleed

## Abstract

Emergency medicine practitioners frequently encounter acute presentations requiring quick, directed treatment to ensure the best patient outcome. Training residents in the appropriate clinical and procedural skills often proves difficult when treating the patient who is critically unwell. Simulation-based medical education is an effective modality that enables teaching around life-threatening medical conditions in a safe space for learners while avoiding adverse complications for patients. Severe gastrointestinal bleeding is one such condition that emergency medicine practitioners would benefit from encountering first in a simulation environment due to its high rate of morbidity and mortality if not quickly managed appropriately.

This report describes a simulated scenario of an undifferentiated patient who is acutely unwell and then becomes hemodynamically unstable secondary to a severe gastrointestinal bleed. Delivery of the case is facilitated by the detailed technical report herein. It contains a stepwise, detailed summary of appropriate learners’ actions and suggestions for learning objectives relating to the case.

## Introduction

Upper gastrointestinal bleeds (UGIB), defined as bleeding that originates proximal to the ligament of Treitz, have an annual incidence between 39 and 172 per 100,000 [1]. In particular, it is more common among males and the elderly [2]. Diagnosis of those patients with UGIB is critical, as many of these will have severe bleeding and require urgent intervention to reduce subsequent morbidity and mortality [1].

The first step in performing an effective, efficient evaluation is to distinguish between a UGIB and lower gastrointestinal bleed (LGIB) as the two differ significantly with respect to management. Upper gastrointestinal bleeding is more common than lower and is also associated with a higher incidence of mortality. The most common causes include peptic ulcer disease (55% incidence, 4% mortality) and esophageal varices (14% incidence, 50% mortality) [3].

However, due to the overlap of symptomatology distinguishing between the two may prove difficult for even experienced physicians. Classical descriptions of initial presentations (such as epigastric pain, altered mental status or systemic shock) are often vague complaints that can be associated with a wide variety of differential diagnoses.

Srygley et al.* *conducted a review of the existing literature to identify which presentations were more indicative of a UGIB versus LGIB: age less than 50 years old; history of passing black, tarry stools; epigastric discomfort; blood-urea-nitrogen (BUN) to creatinine ratio greater than 30; or personal history of UGIB [4].

Next, they also identified symptoms and signs more likely to indicate a UGIB of greater severity: personal history of malignancy or cirrhosis; syncope; tachycardia or hypotension; hemoglobin > 8 g/dL; white blood cell count > 12 x 10^9^/L; or BUN > 90 mg/dL [4]. These general guidelines may help more junior learners differentiate between the two.

Further points on the history frequently discussed as common etiologies of UGIB, such as alcohol, aspirin, and non-steroidal anti-inflammatory drug use, were not useful in clinically distinguishing between the two. Also of note, the absence of tachycardia was the most useful sign in lowering the likelihood of a severe UGIB [4].

Multiple risk stratification models, such as the Glasgow-Blatchford Bleeding, Rockall and AIMS65 scores, have been validated to predict morbidity, mortality and need for urgent intervention by physicians when a suspected UGIB is encountered. Although not a learning objective of this training session, these clinical decision-making tools are available, and have their own associated pitfalls [3,5-6].

Despite improvements in these decision-making models and in preventative measures (such as increased use of proton pump inhibitors (PPIs) among vulnerable patients), mortality from severe UGIB has remained fairly constant at approximately 13% over the past several decades. This is likely explained by an overall aging population with additional medical comorbidities. Therefore, it is important for emergency medicine residents to be familiar with the subtle signs and symptoms of a severe UGIB [2].

Once recognized, it is essential that the physician immediately intervenes and activates the necessary services that can provide definitive therapies. Urgent interventions for the patient with a severe UGIB will begin with volume resuscitation and may include additional steps such as therapeutic endoscopy, blood transfusions, radiological intervention, or surgery [2,4]. Although once considered imperative to the management of acute UGIB, studies have shown that PPIs have no effect on clinical outcomes including mortality, re-bleeding or future surgical need. Despite this fact, they are still often included as part of initial management because of their demonstrated ability to reduce the bleeding time for ulcers and decrease the need for therapeutic endoscopy [7-8].

This case report is designed to train emergency medicine learners in recognizing a UGIB and quickly initiating the appropriate management steps. The learning objectives for this simulation session are:

1. Develop an approach to an undifferentiated, acutely unwell patient 2. Formulate an initial approach to assessment and management of a patient with a suspected upper gastrointestinal bleed 3. Recognize and manage a deteriorating hemodynamically unstable patient with a suspected upper gastrointestinal bleed

The report will follow the Context-Inputs-Process-Product model to present the data compiled [9].

## Technical report

Context

The simulation was designed for the training of emergency medicine residents. However, any off-service learners who are completing Emergency Department (ED) rotations as part of their training, either undergraduate or postgraduate, may benefit from participation as well. It was also designed to occur in a community hospital Emergency Department with some subspecialty backup (e.g.,medicine, general surgery, or critical care).

The scenario is generally run with two to three residents participating, with one as team leader and the others contributing as additional team members. If this simulation is conducted with only one participating resident they should assume the role of team leader and make use of assistance from other confederates who are available.

Inputs

Personnel

Two facilitators were present during the simulation. Facilitators were emergency medicine certified physicians as well as faculty with Memorial University. One observed the scenario, took notes, and compared the learners’ actions to an objective checklist (see Process for more information). The second facilitator ran the scenario and provided prompts to confederates or learners as dictated by the progress of the scenario. The second facilitator also acted as the voice of the consultant if the learner made the call for assistance.

One nurse confederate was used in the simulation. This person was trained in assisting with role playing simulations in order for the learning objectives to be met. They were prepped in advance of the scenario and reviewed the expected course of the simulation with the facilitators prior to the learners’ attempt. They performed tasks for the learners as requested and delivered results/prompts to the learners when instructed to do so by the facilitators. In the case where fewer residents were participating, more confederates may be needed to assist the learners.

Equipment

**Figure 1:**
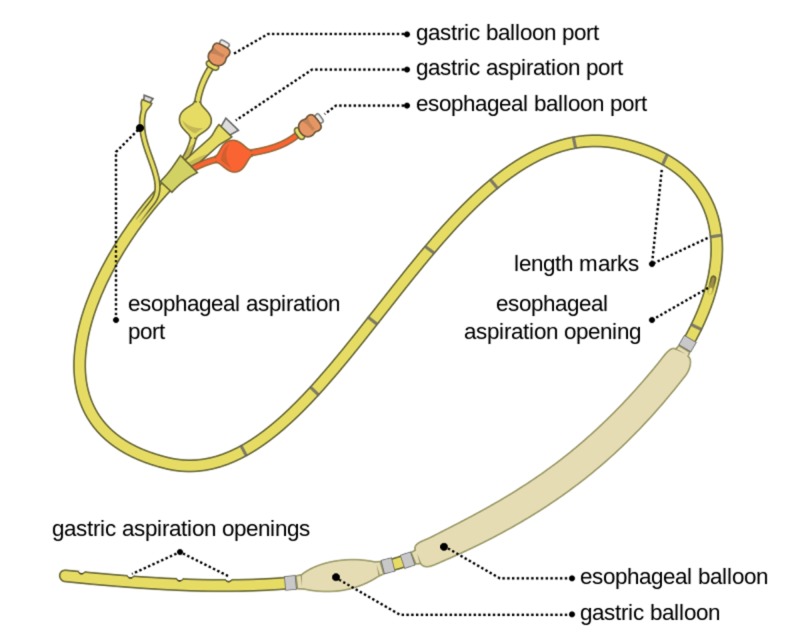
Example of a balloon tamponade device ("Sengstaken-Blakemore© Tube" by Olek Remesz is licensed under CC BY-SA 3.0 via Wikimedia Commons).

The training session was conducted in a simulation lab using a Laerdal SimMan 3G human patient simulator. The lab itself was outfitted with equipment, medications and supplies typical for the resuscitation bay and those needed specifically for this case. This included the following items:

· Advanced cardiac life support cart/defibrillator, with standard medications · Airway cart including oxygenation, intubation supplies & suction · Central line access supplies, task trainer & an available bedside ultrasound · Intravenous (IV) access supplies (including 16- or 18-gauge catheters), 0.9% normal saline & Ringer's lactate solution · Cardiac/oxygen monitors · Available blood products: fresh frozen plasma (FFP) & O-negative packed red blood cells (pRBCs) · Medications: Vitamin K, pantoprazole, octreotide, vasopressors & inotropes (dopamine, norepinephrine, phenylephrine & vasopressin), as well as Rapid Sequence Intubation (RSI) drugs · Foley catheter & glucometer · Sengstaken-Blakemore© tube (or equivalent balloon tamponade device) (See Figure 1 for an example of such a device)

Although high fidelity mannequins are beneficial, if a facility is limited with certain technology, even low fidelity mannequins are an acceptable option for practice and integration into the scenario.

Process

Prior to the session, a stepwise, detailed scenario was generated and submitted to the simulation laboratory technical staff. The mannequin was then programmed accordingly and any necessary materials for the simulation were obtained. The facilitators and confederate reviewed the scenario and ran through the simulation with the facilitators acting in the role of the learners to assess for any problem areas.

Pre-Briefing

A standardized approach is used for our simulation case pre-briefing. This accounts for the fact that learners will vary in their simulation knowledge and experience. It also accounts for the fact that some of the learners in our program have not recently participated in a simulation case. It is designed to clearly establish the structure of the session and review the reasoning behind topic selection; usually a commonly encountered complaint or an uncommon topic about which they must be familiar. Potential limitations of simulation equipment are discussed and the “fiction contract” is reviewed [10].

Pre-Scenario Information

You are the physician(s) working in the ED of your community hospital. A call comes in from the paramedics that they are five minutes away from your ED. They picked up a 65-year-old man from his home after his wife called 9-1-1. He has been progressively more nauseous the past two days, and today seemed very lethargic. Paramedics report he currently has a Glasgow Coma Score (GCS) of 14 and is slightly disoriented. His main complaint currently is moderate epigastric discomfort and nausea. He appears unwell and is quite diaphoretic.

The Simulation

Table 1 summarizes historical, physical and investigative findings that the learners encountered as they perform their initial assessment and ordered initial lab work.

**Table 1:** Summary of initial patient findings upon presentation to the Emergency Department to guide completion of Learning Objective #1. BID = Twice daily; GI = Gastrointestinal; HEENT = Head-Eyes-Ears-Nose-Throat; PO = Taken orally.

A Summary of Pertinent Patient Findings	
Pertinent Findings on History	
Identification	Joe Summers, 65-year-old male, wishes for full resuscitation
History of Presenting Illness	- 2/7 nauseous, emesis x4 today but wife and patient never noticed colour/quality - Disoriented/confused starting today - Shortness of breath on exertion x 2/7 - Epigastric pain (sharp, non-radiating) has been increasing in intensity (4/10 à 7/10 now) - History of dark stools in recent weeks. Last bowel movement today was loose and dark as well
Past Medical & Surgical History	- Hypertension - Dyslipidemia - Bilateral knee osteoarthritis - Chronic back pain - Appendectomy at 19 years old
Medications	- Rosuvastatin 20 mg PO daily - Naproxen 500 mg PO BID - Hydrochlorothiazide 12.5 mg PO daily
Allergies	No known drug allergies
Family History	No history of cardiac or GI diseases
Social History	- Works as a chef, married with no children - Drinks two beer per day in the evening, 40 pack year history of tobacco use, occasional marijuana smoker, no illicit drugs
Pertinent Findings on Physical Exam	
Initial Vitals	Heart rate 110 (normal sinus rhythm), Blood pressure 95/60, Respiratory rate 16, Oxygen Saturation (SpO2) 96% room air, Temp 37.0°C, Glucose 4.6 mmol/L
General	Alert & oriented x3, lethargic, pale, diaphoretic
HEENT	Pale conjunctiva
Neurological	Cranial nerves grossly intact, patient able to move limbs x4 but unsteady gait
Cardiovascular	Tachycardia, radial pulses weak but regular, heart sounds normal without murmur, pedal pulses barely palpable, no lower limb edema
Respiratory	Good air entry bilaterally
Abdominal	Hyperactive bowel sounds, pain with percussion and palpation in upper quadrants with maximum at epigastrium, no peritonitis or guarding
Genitourinary	Digital rectal exam confirms presence of melena
Pertinent Findings from Investigations Test Name, Value (Normal Range) – Bold indicates abnormal value	
Complete Blood Count (CBC)	- Hemoglobin 114 (140 – 180 g/L) - Mean Corpuscular Volume 98.2 (80 – 97) - Leukocytes 13.2 (4.8 – 10.8 x 10^9^/L) - Neutrophils 9.3 (2.0 – 7.5 x 10^9^/L) - Platelets 120 (130 – 400 x 10^9^/L)
Electrolytes & Renal Function	- Sodium 128 (135 – 145 mmol/L) - Potassium 3.3 (3.5 – 5 mmol/L) - Chloride 101 (95 – 110 mmol/L) - Glucose 4.5 (2.5 – 7.8 mmol/L) - Urea 10.1 (3 – 7 mmol/L) - Creatinine 105 (54 – 113 mmol/L) - Estimated Glomerular Filtration Rate (eGFR) 62 (>60)
Liver Enzymes & Associated Markers	- Aspartate Aminotransferase (AST) 84 (0 – 37 IU/L) - Alanine Aminotransferase (ALT) 60 (0 – 55 IU/L) - Alkaline Phosphatase (ALP) 140 (40 – 150 IU/L) - Amylase 110 (25 – 125 IU/L) - Bilirubin 24 (3.4 – 20.5 umol/L) - Albumin 23 (35 – 52 g/L)
Toxicology Screen	- Acetaminophen negative - Ethanol 10 (0 – 10.85 mmol/L) - Salicylate negative
Coagulation Studies	- International Normalized Ratio (INR) 1.35 (0.80 – 1.20) - Activated partial thromboplastin time (aPTT) 30.1 (26.1 – 35.3 sec)

Table 2 describes a stepwise, detailed scenario that was used by facilitators and technical staff to run the simulation. The authors suggest that inclusion of points listed under Objective #3 in the table be reserved for the advanced learner with some flexibility built into the case as dictated by the performance of the learner. As indicated in the table, pulseless electrical activity (PEA) arrest with or without successful resuscitation outcomes can be an optional end to the case. The inclusion of death as a potential outcome for the case should be predetermined by the case facilitators.

**Table 2:** A stepwise, detailed summary of the learners’ expected actions for each learning objective and the associated patient findings/outcome. ACLS = Advanced cardiac life support; BP = Blood pressure; CBC = Complete Blood Count; ECG = Electrocardiogram; GCS = Glasgow Coma Score; HR = Heart rate; IV = Intravenous; LBC = Electrolytes, Blood Urea Nitrogen, Creatinine; O2 = Oxygen; PEA = Pulseless electrical activity; pRBC = Packed red blood cells; RA = Room air; RR = Respiratory rate; RSI = Rapid sequence induction; ROSC = Return of spontaneous circulation; SpO2 = Oxygen Saturation; T = Temperature; T&S = Type and Screen; XM = Crossmatch blood.

Summary of Learning Objectives and Expected Learner Actions	
Learning Objective #1: Develop an approach to the undifferentiated, acutely unwell patient.	
Expected Actions	Pertinent Findings/Outcome
Obtain history: from the patient & collateral history	Initial vital signs: HR 100 (normal sinus rhythm) // BP 95/60, RR 16 // SpO2 96% RA // T 37.0°C // Glucose 4.6 mmol/L
Note vital signs	
Perform focused physical exam	
Order cardiac monitoring	
Obtain IV access – 2 large bore IVs	
Begin oxygen administration as necessary	
Request repeat set of vital signs	
Learning Objective #2: Formulate an initial approach to the assessment and management of a patient with a suspected upper gastrointestinal bleed	
Expected Actions	Pertinent Findings/Outcome
Order bloodwork (CBC, LBC, liver enzymes, coagulation factors, lactate, T&S, XM)	ECG & Bedside ultrasound results are normal and are "retrieved" quickly for learners See Table 1 for bloodwork findings which take longer to "retrieve"
Investigations: ECG +/- bedside ultrasound	
Administration of IV proton pump inhibitor	
Fluid resuscitation & consider transfusion of O-negative blood	If adequate (minimum 1 L bolus followed by continuous infusion and initiation of pRBC transfusion) go to 2A If inadequate go to 2B
Verbalize other treatment options depending on patient condition, history, comorbidities, and suspected etiology: Vitamin K, Octreotide, Fresh Frozen Plasma, Octaplex, antibiotics (Ceftriaxone), and/or erythromycin	
Stage 2A — Adequate Initial Volume Resuscitation — Vitals: HR 94 // BP 102/66	
Adequate volume resuscitation	Patient's vital signs transiently improve before going to Learning Objective #3 ** For junior learners: if all actions to this point have been completed/verbalized, scenario can end here **
Stage 2B — Inadequate Volume Resuscitation — Vitals: HR 122 and BP 85/60	
Learners make no further resuscitation attempts after a period of time. Nurse prompt as needed: e.g., “He looks like he is getting sicker”	Go to Learning Objective #3
Learning Objective #3: Recognize and manage a deteriorating hemodynamically unstable patient with a suspected upper gastrointestinal bleed (Advanced Learner) Patient Action: Vomits large amount of frank, red blood (this deterioration occurs in any case, whether or not proper actions taken to this point). Reassessed Vitals : HR 150 // BP 70/40 // T 37.0°C // RR 6 // SpO2 90–92% 4L O2 via nasal prongs // GCS 7	
Expected Actions	Pertinent Findings/Outcome
Appropriate RSI intubation	If all actions completed go to 3A If actions missed go to 3B
Central line inserted correctly (optional)	
Administer the following as appropriate to details of the case: Vitamin K, Octreotide, Fresh Frozen Plasma, Octaplex, antibiotics (Ceftriaxone), and/or erythromycin	
Verbalize consideration of balloon tamponade device	
Activation of massive transfusion protocol	
Consult appropriate disposition service	
Stage 3A — Completion of all Expected Actions	
End Scenario – Vitals HR 95 // BP 105/64 // T 37.0°C // RR 12 // SpO2 96% (intubated)	
Patient care taken over by specialist arrival or transfer to critical care unit	
Stage 3B — Failure to Complete all Learning Objective/Expected Actions Above	
Nurse prompt e.g., “his blood pressure is still low” OR “he is getting more tachycardic”	Learner takes appropriate actions above. Vitals stabilize. Go to 3A.
No further actions by learner	If no actions taken or inadequate in response to nurse prompt, patient remains in critical condition with vitals deteriorating. End scenario or go to Optional Objective
OPTIONAL Objective – PEA arrest if failure to take appropriate earlier actions	
Appropriate resuscitation (using ACLS guidelines)	Patient achieves ROSC. End scenario.
Inappropriate/inadequate resuscitation (using ACLS guidelines)	Failure to resuscitate. End scenario

Table 3 outlines a number of modifiers that can be used to enhance the difficulty of the gastrointestinal (GI) bleed simulation case. This includes examples of historical and physical exam findings that challenge the learner to think beyond standard therapies and interventions. The ability to include procedural integration in the case will depend on the desired objectives of the case and upon resource availability with respect to mannequin capability and access to appropriate task trainers.

**Table 3:** Suggested case modifiers utilized to alter the difficulty of the simulation. NSAIDs = Non-steroidal anti-inflammatory drugs; BUN = Blood urea nitrogen; INR = International normalized ratio.

FEATURE	MODIFIER & COMMENTS
Simulated Patient Confederate(s)	
Family member	Knowledgeable & helpful
Friend	Loud, interfering & little knowledge
Historical Findings	
Patient alertness	Alert, knows medications & history
	Altered level of consciousness. Not helpful regarding medications or medical history & must use alternate sources - Medical bracelet - Electronic medical records - Collateral history
Medications	Beta-blockers (effect on heart rate)
	Anticoagulants (effect on bleeding): - NSAIDs - Warfarin - Direct oral anticoagulants
Physical Examination Findings	
Heart rate & rhythm	Sinus tachycardia (junior learners)
	Atrial fibrillation on monitor (A clue to patient potentially being on anticoagulants)
	No tachycardia (senior learner) - e.g. patient on beta blocker
Laboratory data	
Abnormal values (junior learners)	May include the presence or absence of upper gastrointestinal bleed such as a low hemoglobin or increased BUN
Normal values (senior learners)	Normal lab values for hemoglobin and BUN; equivocal INR results
Procedural Integration (advanced learners) - either on the mannequin or a separate task trainer	
Intubation	
Central line placement	McNeil et al. provide a description of appropriate central venous catheter insertion [11].
Balloon tamponade device placement	Weingart has produced a video and tutorial demonstrating the proper insertion of such a device [12].
Simulation Outcome	
Death as a potential endpoint	For the advanced learner and facilitators should decide beforehand if this will be a specific objective and potential outcome.

Debriefing & Post-Scenario Didactics

Following the conclusion of the scenario, facilitators and learners participated in a formal debriefing session. This part of the simulation provided a confidential environment for discussion case details and the pre-determined learning objectives. Care was taken during the debriefing to ensure that the number of instructors was limited such that the instructor-to-learner approximates a 1:1 ratio. Multiple debriefing models have been validated and facilitator preference usually dictates which they would like to utilize [13-15]. Simulated patients and confederates participating in the case attended the beginning of the debriefing session to provide and receive feedback from these individuals, usually with respect to non-medical expert features of the case.

Didactic instruction is integrated to provide further teaching on select topics relating to the objectives of the case. This method of incorporating teaching following the simulation has been shown to be superior to pre-simulation instruction alone [16].

The content of the debriefing session is generally guided by the predetermined objectives for the case. Depending on how the scenario progresses, modifications can be made to ensure that unexpected, while important, issues are addressed for the learners. These could include additional topics, controversy or conflict. In this session, the junior residents were instructed primarily on an approach to the undifferentiated patient who presents acutely unwell to the ED, and the initial approach to acute UGIB management including fluid resuscitation, early medication administration, and appropriate calls to specialty backup. Awareness of next steps depending on specifics of the scenario is also important and will usually be discussed in limited detail with junior learners (such as how to handle complications or a deteriorating patient despite appropriate initial management). In contrast, more advanced learners will perform the early assessment and management and then proceed to advanced measures when the patient responds poorly or in the setting of further deterioration.

Modifiers and optional procedural integration noted for the case can be applied in a variety of ways to alter difficulty and allow repeated use of the case, stressing different points at each encounter. Examples of historical modifiers could include: the non-steroidal anti-inflammatory drug (NSAID) associated upper GI bleed; the patient on Warfarin (or other anticoagulation medication) for atrial fibrillation; the heavy drinker with undiagnosed cirrhosis; or a patient with a mechanical valve, on anticoagulants, presenting with a new GI bleed. The post-scenario didactic session is a particularly valuable time for discussion on specific advance interventions such as the administration of blood products like Octaplex©, antibiotics in the context of cirrhosis, and the use of a Sengstaken-Blakemore© tube (or similar balloon tamponade device) [12, 17-18].

**Figure 2:**
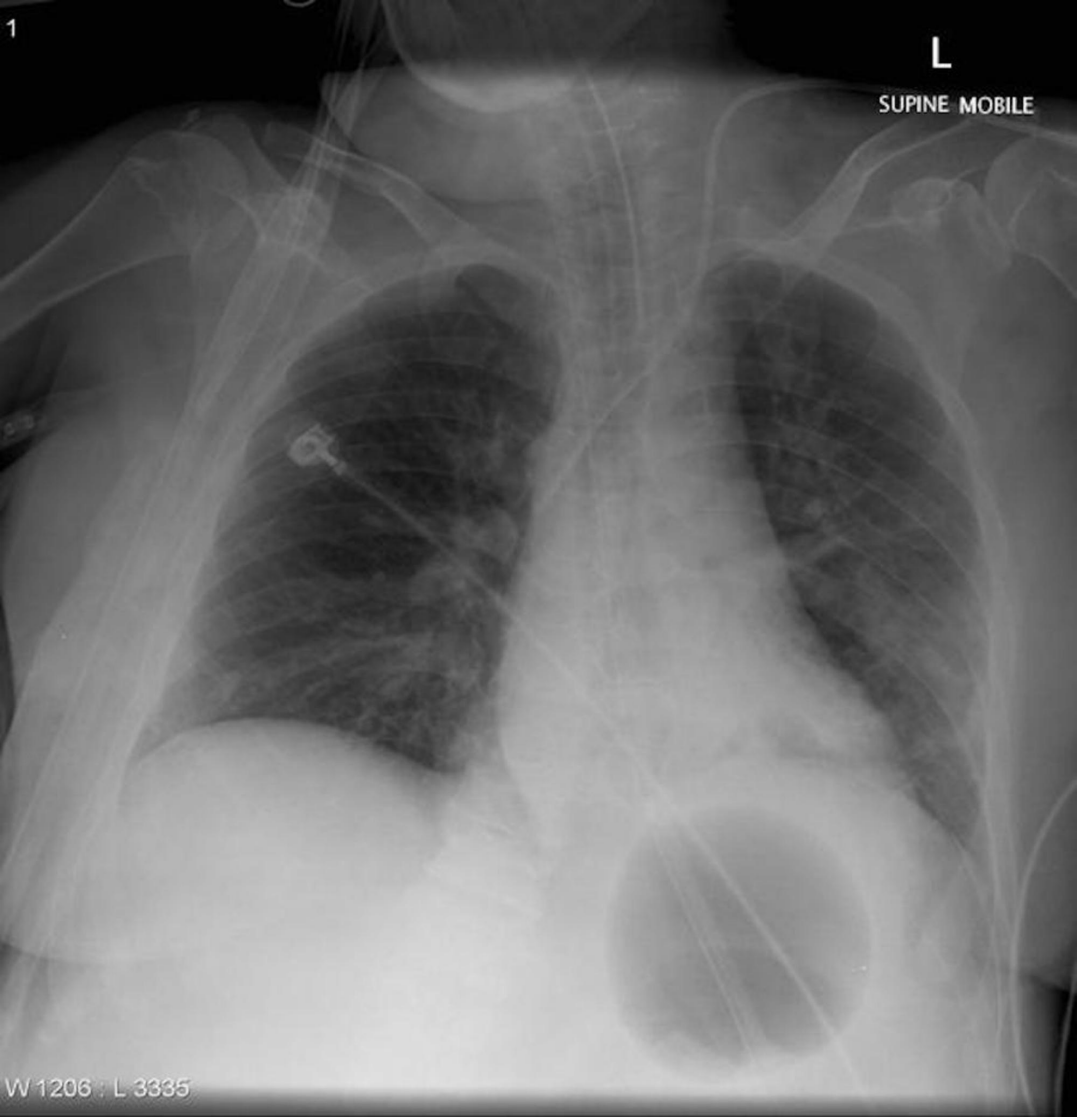
A supine chest X-ray demonstrating correct placement of a Sengstaken-Blakemore© tube (Case courtesy of A.Prof Frank Gaillard, Radiopaedia.org, rID: 12118).

Insertion of the Sengstaken-Blakemore© tube, while not a part of this simulation, is a useful adjunct in scenarios where the UGIB is unable to be controlled and therapeutic endoscopy is not immediately available. While multiple studies have shown that it is not without risk (significant re-bleeding rates, low esophageal rupture rates) it does perform well as a temporizing measure to bridge the patient to definite therapy [18]. Weingart has produced a video and tutorial demonstrating the proper insertion of such a device [12]. Figure 2 demonstrates a chest radiograph of in situ Sengstaken-Blakemore© tube.

The GI bleed scenario also presents a valuable opportunity to discuss challenges that may be encountered with the intubation required in Learning Objective #3 of the case.

Recently, there has been a fair amount of discussion about the benefits of including death as an outcome in simulation-based medical education. Given that unexpected death is a reality in emergency medicine, there exists controversy over whether to allow the simulator to die when death is not a predefined learning objective. Some have speculated that the unforeseen death of a simulator can cause undue psychological harm to the student, whereas others believe that it enhances their learning [19-20]. As indicated in the table, a PEA arrest with or without successful resuscitation outcomes can be an optional end to the case. Alternatively, if facilitators deem this step inappropriate for the learners, the simulation could simply be stopped prior to that event.

Product

The expected outcomes for each participant are outlined by the learning objectives for this simulation:

1.** **Develop an approach to the undifferentiated acutely unwell patient. 2.** **Formulate an initial approach to the assessment and management of a patient with a suspected upper gastrointestinal bleed. 3.** **Recognize and manage a deteriorating hemodynamically unstable patient with a suspected upper gastrointestinal bleed.

Additional objectives may be included by particular facilitators depending on the overall goals of the session and topics of the post-didactic session. We generally try to have approximately three objectives with an effort to integrate a non-medical expert objective into the case when possible.

## Discussion

The ability to manage acute upper gastrointestinal bleeding is crucial for anyone practicing acute care medicine. The simultaneous assessment, investigation and management are challenging even for the experienced practitioner. Simulation creates an environment that permits learners to make real-life decisions without the possibility of adverse patient outcomes. In the context of a UGIB, and the associated high mortality rate, simulation may be a valuable teaching modality to facilitate essential skill development.

The development of the case scenario using a stepwise algorithm allows the simulation to unfold according to decisions made by trainees. Having a facilitator complete the run-through in advance ensures both that the case is of reasonable difficulty for the learner and enables instructors to address any limitations of the scenario. Finally, the use of a formal debriefing coupled with a post-scenario didactic session allows collaborative identification of knowledge gaps and process errors that arise during the simulation.

## Conclusions

Teaching emergency medicine residents to identify and manage an acute UGIB through the use of simulation promises to be of value. It permits learner development in an environment safe for both patients and learners. The case also presents flexibility with respect to the degree of difficulty appropriate for each individual group of learners. This report describes a template that institutions may use to train their own learners in these skills. A stepwise approach for the simulation is developed to facilitate the execution of a scenario and an integrated teaching session incorporating simulation and didactics with components of debriefing is described herein.
